# Performance Assessment of Object Detection Models Trained with Synthetic Data: A Case Study on Electrical Equipment Detection

**DOI:** 10.3390/s24134219

**Published:** 2024-06-28

**Authors:** David O. Santos, Jugurta Montalvão, Charles A. C. Araujo, Ulisses D. E. S. Lebre, Tarso V. Ferreira, Eduardo O. Freire

**Affiliations:** 1 Department of Electrical Engineering, Federal University of Campina Grande, Campina Grande 58401-490, Brazil; 2 Department of Electrical Engineering, Federal University of Sergipe, São Cristóvão 49100-000, Brazil; jmontalvao@academico.ufs.br (J.M.); tarso@academico.ufs.br (T.V.F.); efreire@academico.ufs.br (E.O.F.); 3 Electrical Operation, Eneva S.A., Barra dos Coqueiros 49140-000, Brazil; charles.cordeiro@eneva.com.br (C.A.C.A.); ulisses.lebre@eneva.com.br (U.D.E.S.L.); 4 National Council of Scientific and Technical Research—CONICET, Godoy Cruz, Buenos Aires 2290, Argentina

**Keywords:** electrical equipment, infrared spectrum, machine vision, object detection, synthetic data, Viola–Jones, visible spectrum, YOLO

## Abstract

This paper explores a data augmentation approach for images of rigid bodies, particularly focusing on electrical equipment and analogous industrial objects. By leveraging manufacturer-provided datasheets containing precise equipment dimensions, we employed straightforward algorithms to generate synthetic images, permitting the expansion of the training dataset from a potentially unlimited viewpoint. In scenarios lacking genuine target images, we conducted a case study using two well-known detectors, representing two machine-learning paradigms: the Viola–Jones (VJ) and You Only Look Once (YOLO) detectors, trained exclusively on datasets featuring synthetic images as the positive examples of the target equipment, namely lightning rods and potential transformers. Performances of both detectors were assessed using real images in both visible and infrared spectra. YOLO consistently demonstrates $F_{1}$ scores below 26% in both spectra, while VJ’s scores lie in the interval from 38% to 61%. This performance discrepancy is discussed in view of paradigms’ strengths and weaknesses, whereas the relatively high scores of at least one detector are taken as empirical evidence in favor of the proposed data augmentation approach.

## 1. Introduction

The problem of simultaneous localization and classification of objects in images is referred to as object detection. Localization means the capacity to draw a bounding box around each object of interest in the image, whereas classification means assigning to each bounding box a class label (for the localized object) [1]. As reported in [2], there are factors that complicate these two tasks, like large variations in viewpoints, poses, occlusions, and lighting conditions.

Despite these difficulties, there have already been remarkable achievements in object detection [3]. An example is the Viola–Jones (VJ) detector, which was the first algorithm that obtained high accuracy in the face detection problem in real time [4,5]. Due to advances in neural network architectures, the computational power of modern computers, and access to huge databases of images, detectors based on Convolutional Neural Networks (CNNs) have been successfully applied in the detection of different types of objects [6,7,8].

On the other hand, if databases used in the training of neural networks are not large enough, then the performance of CNN based detectors may be poor, which is a well-known problem of model over-fitting that commonly occurs as a consequence of limitations in manually acquiring and labeling large amounts of images. Indeed, very large deep neural models have many degrees of freedom (free parameters), which demands an equally large amount of labeled examples to mitigate the risks of over-fitting [9].

Among the proposed approaches to deal with over-fitting are the learning transfer [10] and pre-training [11] techniques, which use as initial neural network parameters those of another network that has been trained on another dataset. An alternative approach is data augmentation which, unlike the previous ones, deals with the problem of over-fitting at its root, the scarcity of data in the training base [6].

In the taxonomy presented in [6], the image data augmentation approach is divided into two branches. The first branch includes techniques that create new images from basic manipulations of existing ones, like color space and geometric transformations. A problem with the approaches included in this branch is that they need initial data, which may not be available in some cases. The other branch includes techniques based on deep learning, such as Generative Adversarial Networks, in which a generative neural network is designed to create new images in such a way that a discriminating neural network is unable to differentiate between created and real images [12,13]. Another approach that also deals with the over-fitting problem, but that does not depend on the previous existence of data is image synthesis [14]. In this approach, images are generated from scratch by computer graphics algorithms in a process known as rendering. This process uses descriptive models that involve the geometry and sometimes textures of the target objects such as technical drawings, CAD models, and mathematical equations.

The use of synthesized images is an attractive approach in object detection problems where the target object has a rigid body (which is thus easily synthesized because the distance between any two points of it is constant), but real images of them are scarce, as is the case with thermal images of electrical power equipment. Additionally, during their synthesis, the images can be automatically labeled.

Indeed, visual inspection of electrical equipment in power plants can prevent economic losses caused by power outages. These inspections are usually performed through infrared images, in which temperature distributions can be measured and used to detect early insulation failure, overloading, and inefficient operation [15,16,17,18,19]. However, other kinds of images can also be used, including images taken in the visible spectrum. In all cases, any such inspection automation must rely on the explicit or implicit equipment localization in the image, along with its classification [20], which makes it a well-suited application for the data augmentation approach studied in this work.

The primary focus of this paper is to present a case study conducted under the hypothetic restriction that images from the target objects are not available at all, as in the early stage of an industrial plant design, where only detailed geometric information about the object is available. The study focuses on the performance assessment of two well-established machine learning approaches belonging to different paradigms when all positive training examples are synthetic images. Additionally, training datasets also included non-synthetic instances of non-target images, all preprocessed and converted to black and white (binarized). The binarization process of these images discards most information not related to the geometry of the targeted rigid objects in images, and allows the use of the same detector for both visible and infrared spectra, as studied in this work. Another suitable consequence of systematic image binarization is its simplification of the synthetic image rendering process. In this case, only the geometric shapes of the devices are needed, eliminating the necessity for precise colors and textures, which are considered to be less relevant information for detecting well-defined rigid bodies in images.

The study focuses on two distinct classes of power electrical equipment images: lightning rods and potential transformers, which were arbitrarily selected for examination. The chosen learning machines for this investigation are the Viola–Jones and YOLO detectors. The Viola–Jones detector was selected due to its expandability and efficiency. The YOLO detector was also selected as a prominent representative of the last trend in the connectionist paradigm, deep learning, which combines high accuracy with runtime speed [21]. Subsequently, both detectors were tested using non-synthetic images from the GImpSI database (Gestão dos Impactos da Salinidade em Isolamentos), encompassing both the visible and infrared spectra. In addition to lightning rods and potential transformers, the GImpSI database includes images containing other electrical equipment such as transformers, current transformers, circuit breakers, disconnect switches, and pedestal insulators.

Although we assume that no images of the target object are initially available, we also assume that the user can specify the angles at which future images will be acquired. In our case, the real images from the GImpsi dataset serve as a simulation of the future data that the detector will use. Therefore, we generated synthetic images specifically for the angles that are anticipated to be used, ensuring that our model is trained and evaluated under realistic conditions.

The aim of this study is to obtain a better understanding of the effects of exclusively using synthetic images as positive examples for training, thus addressing situations where no real images of the target objects are accessible during the training phase.

This paper is organized as follows: in Section 2, a brief literature review is conducted on the use of synthetic images in training datasets; in Section 3, the computer graphics approach used to synthesize images of power electrical devices is described; Section 4 contains the description of both the VJ and YOLO detectors; in Section 5, the databases used to train and test the detectors are presented; the experiments conducted using the synthesized images and the detectors are displayed and discussed in Section 6; in Section 7, the conclusions of the work are presented.

## 2. Related Work

The use of synthetic data in pattern recognition tasks such as object detection and segmentation has emerged as a solution to three problems: the scarcity of data, the storage of large amounts of data, and their laborious manual labeling. The scarcity is solved because computer graphics techniques can generate the desired amount of images. In addition, these techniques also solve the labeling problem, since when rendering each object, its class and location are already known. Storage can also be tackled because synthetic images can be rendered and immediately used for training, after which they can be discarded, freeing up memory space [14]. Because of these benefits, synthetic data is used for many types of objects. For example, in [22], several approaches based on CNNs were trained only with synthetic data to detect vehicles, pedestrians, and animals. The validation results with synthetic data were similar to those obtained with real data.

Another context in which synthetic images are used is the segmentation of table objects, to help robotic systems to grab them. In [23], experiments were performed with a CNN that was trained with synthetic and real data to segment table objects with results that indicated that performance is positively correlated with the number of synthetic images.

Performance improvement was also noticed in the experiments carried out in [24], which also indicated that supplementing a database with synthetic images is better than other data augmentation approaches. The task in which these experiments were performed was the detection of flaws in wafer mapping images, to identify irregularities in semiconductor manufacturing processes.

Synthetic images are also useful in infrequently occurring contexts. For example, in [25], a VJ detector was trained in the task of detecting lifeboats using fully synthetic data, which were generated through graphical simulations of the 3D model of a lifeboat and sea waves. To validate the detector performance, images taken from the rescue operation of the Russian fishing trawler “Dalniy Vostok” in 2015 were used, with recall and precision rates of 89% and 75%, respectively.

In the healthcare domain, synthetic data has been explored in many works as a solution to challenges involving privacy risks and regulations that restrict researchers’ access to patient data [26]. A comprehensive review of the use of synthetic medical images is provided in [27]. This review covers important applications, including magnetic resonance imaging and computerized tomography.

Synthetic images have been explored in various other applications, such as insect detection [28], spacecraft fly-by scenarios [29], hand localization in industrial settings [30], and industrial object detection with pose estimation [31].

It should be further highlighted that an interesting source of synthetic images are electronic games, since their developers have been striving to make virtual scenarios increasingly realistic. However, famous games like Grand Theft Auto (GTA) do not usually support labeling automation. As a solution, researchers have been developing their own virtual worlds through the Unreal Engine (UE4) platform, since there are extensions to it that automate data generation and its labeling, as through the open source project UnrealCV [32].

In this work, however, synthetic images are created from scratch, by means of straightforward programming codes corresponding to what is described in Section 3. This approach was preferred because synthetic images used here are simple black-and-white renderings of targeted devices projection, in a limited range of poses, and the from-scratch approach allowed more control of this rendering process.

## 3. Synthetic Data Generation

For reproduction purposes, the rendering approach used in this work is explained in this section, which is a simplified version of the rasterization method explained in [33]. The methodology consists of modeling objects in 3D space as a finite set of triangles, projecting the triangles on a 2D plane, which is eventually converted into a black-and-white image.

### 3.1. Rendering Process of a Point

**Figure 1:**
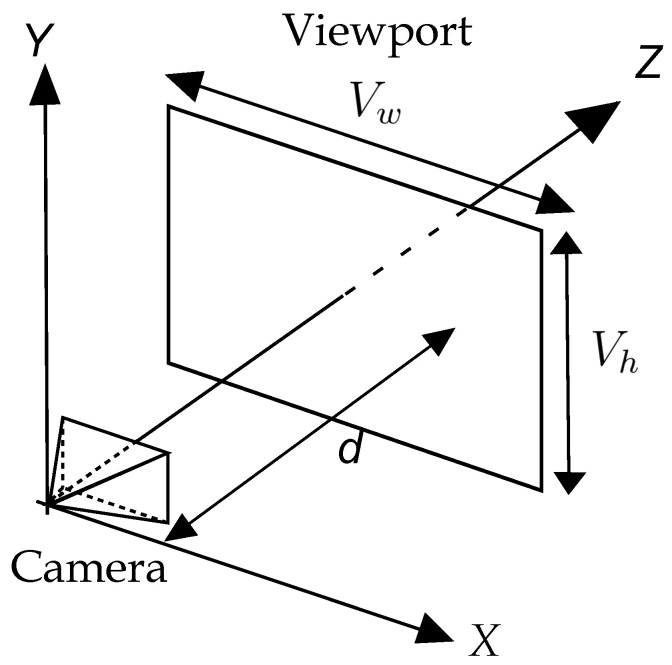
Camera and viewport, based on [33].

Two entities are defined for the rendering process. The first one is the camera which has attributes such as observer position and orientation, and the second is the viewport, which is a three-dimensional representation of the canvas [33]. These two entities are shown in Figure 1. The camera is located at the origin of the coordinate system with an orientation equal to the positive Z-axis, and the viewport, with dimensions $V_{w}\times V_{h}$, is located at a distance *d* from the camera along the Z-axis.

**Figure 2:**
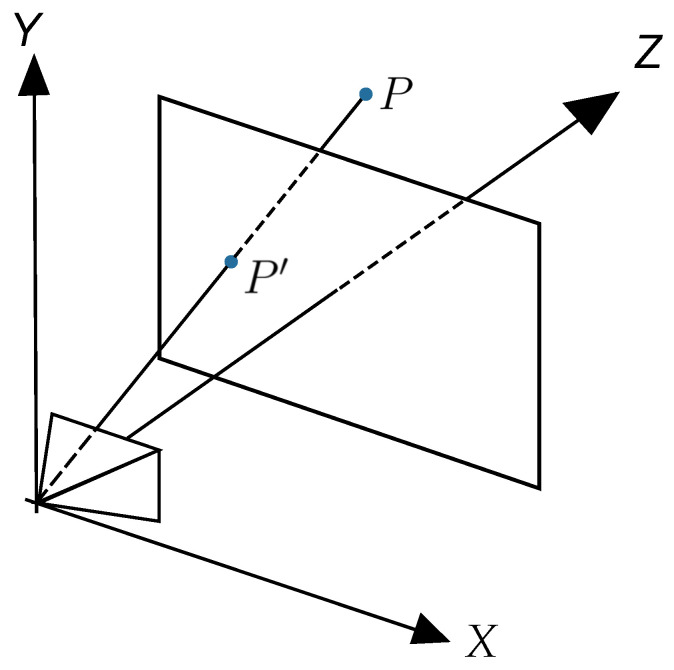
Projecting a point P onto the viewport, based on [33].

Thus, the rendering process involves converting into an image everything that the camera “sees” through the viewport. For example, the rendering process of point *P*, shown in Figure 2, is the calculation of $P^{\prime }$ coordinates, and its representation as a pixel on the canvas.

Let $P=(P_{x},P_{y},P_{z})$, $P^{\prime }=\left(P_{x}^{\prime },P_{y}^{\prime },P_{z}^{\prime }\right)$ and *d* be the distance from the camera to the viewport, so (1) and (2) can be derived from the relationships of similar triangles:

(1) $$P_{x}^{\prime }=\frac{P_{x}\cdot d}{P_{z}},$$

(2) $$P_{y}^{\prime }=\frac{P_{y}\cdot d}{P_{y}}.$$

To complete the rendering process of point *P*, the values of ($P_{x}^{\prime },P_{y}^{\prime }$) must be converted into coordinates (x,y) of the canvas, which are given in pixels, whereas the coordinates of the viewport are given in meters. Furthermore, as shown in Figure 1, the XY plan is parallel to the viewport, and the intersection between the Z-axis and the viewport is its center, but the origin of the canvas coordinate system is in the upper left corner, and its Y-axis is in the opposite direction to the viewport’s Y-axis. Thus, conversion from ($P_{x}^{\prime },P_{y}^{\prime }$) to (x,y) requires a scale adjustment, a translation, and a change in the direction of coordinate *y*. These operations are summarized in (3) and (4):

(3) $$x=\frac{C_{w}\left(P_{x}^{\prime }+0.5V_{w}\right)}{V_{w}},$$

(4) $$y=\frac{C_{h}\left(-P_{y}^{\prime }+0.5V_{h}\right)}{V_{h}},$$

where $C_{w}$ and $C_{h}$ are the width and height of the canvas, respectively.

### 3.2. Rendering Process of a Triangle

**Figure 3:**
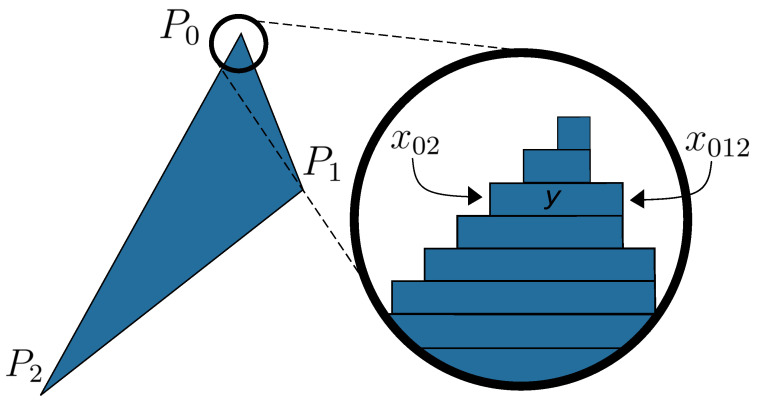
Drawing of a filled triangle using horizontal line segments, based on [33].

The first step in the rendering process of a triangle is the rendering of its three vertices. Once the positions of the three points on the canvas are known, it is possible to draw the triangle defined by them. The drawing of a filled triangle can be decomposed into several drawings of horizontal line segments, as shown in Figure 3. Therefore, a methodology to draw a triangle is to calculate the values of the two extremities, $x_{02}$ and $x_{012}$, for each value of *y*, and then paint the pixels from one extremity to the other.

The list of values for $x_{02}$ and $x_{012}$ can be obtained using the Interpolate algorithm shown in Algorithm 1. This algorithm generates a list of *x* values between point $P_{a}$ and point $P_{b}$. Given the three vertices of the triangle $P_{0}$, $P_{1}$, and $P_{2}$, and assuming $y_{0}\leq y_{1}\leq y_{2}$, then $x_{02}$ is the output of Interpolate($P_{0},P_{2}$), whereas $x_{012}$ is the concatenation of the results of Interpolate($P_{0},P_{1}$) and Interpolate($P_{1},P_{2}$), except for the removal of $x_{1}$ from one of the two results. Furthermore, the values of *y* are from $y_{0}$ to $y_{2}$ with a step equal to 1.
**Algorithm 1** Interpolate**Require:** $P_{a}=\left(x_{a},y_{a}\right),P_{b}=\left(x_{b},y_{b}\right)$**Ensure:** list 1: list = EmptyList() 2: **if** $y_{a}==y_{b}$ **then** 3:   list.append($x_{a}$) 4: **else** 5:    $a=\left(x_{b}-x_{a}\right)/\left(y_{b}-y_{a}\right)$ 6:    $b=x_{a}-a\times y_{a}$ 7:    **for** $i=y_{a}$ to $y_{b}$ **do** 8:      x=a×i+b 9:      list.append(*x*)10:    **end for**11: **end if**

In the process of rendering a complex object, it must be modeled by a finite set of triangles, each of which must be rendered. This procedure can require even more care and processing if the object has more than one color. However, in this work, all objects have the same color, since the geometric information is sufficient to differentiate power electrical equipment. In [33], there are details on how to implement the rasterization approach when objects have multiple colors.

**Figure 4:**
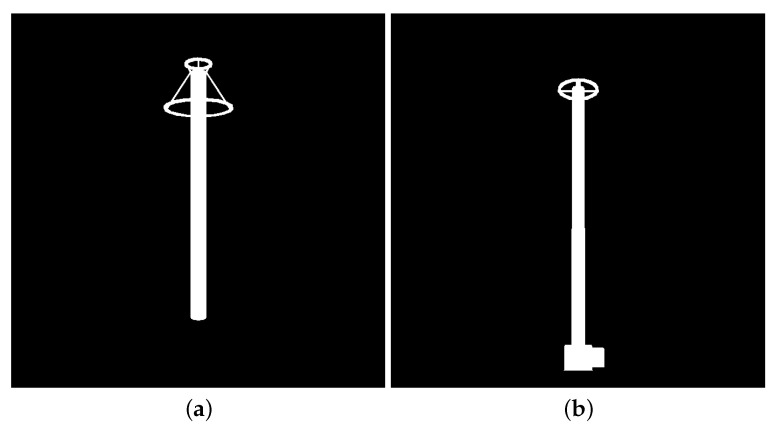
Examples of synthetic images of each target equipment. (**a**) Lightning rod, (**b**) Potential transformer.

With the described approach and the geometric descriptions of each equipment targeted in this paper, it is possible to synthesize images with the desired scale and orientation of each of the equipment. In Figure 4, examples of synthetic images of each equipment are shown, which were rendered using the described methodology.

## 4. Detectors

This section provides brief explanations of the architecture of both the VJ and the YOLO detectors, along with a description of the training setup.

### 4.1. Viola–Jones (VJ)

Proposed in the early 2000s, the VJ detector was the first method to detect human faces in real time without any restriction [4,5]. At the time, it was 10–100 times faster than any other approach with similar accuracy [3]. Other examples of objects in which VJ was successfully applied include vehicles [34], pedestrians [35], license plates [36], hands [37], and birds [38].

The architecture of the VJ detector makes it a sliding window method, as it scans rectangular regions of the image, indicating whether the target object is present or not. In other words, it behaves just like a classifier for each possible rectangular region of the scanned image. In order for the scanning to be performed quickly, the method uses Haar features and the concept of the integral image. Together, these two VJ features allow the method to calculate the difference between rectangular regions of the images with a few basic addition and subtraction operations. A third concept that makes the method even faster is cascade classification, which replaces a single classifier with several cascade classifiers, aiming to discard most of the negative examples at the beginning of the cascade, whereas only a few negative examples, similar to the positive ones, reach the final stages. In addition, the AdaBoost algorithm is used to combine the classifiers in the cascade. The version of the algorithm presented and used in this paper is the original approach proposed by Viola and Jones in [5]. However, it should be emphasized that there have been improvements proposed for the VJ detector in the literature. In [39], the history of its development and modifications is reported.

In this work, the VJ models were designed such that each strong classifier achieves a false positive rate of at most 0.35 and a detection rate of at least 0.99. Additionally, each strong classifier was trained using 2000 synthetic examples and a maximum of 4000 sub-windows of negative examples.

### 4.2. YOLO

The YOLO is a detector based on CNN and is referred to in the literature as a one-stage detector, which means that it applies a single neural network to the full image [3]. This characteristic has made it a fast detector, even for multi-class object detection, when compared to other detectors based on CNN.

The first version of YOLO was proposed in [21], and it has received multiple updates to improve its detection accuracy [40,41]. The latest version is YOLOv8, and a comprehensive review of all of the updates is reported in [42]. The version employed in this paper is the open-source YOLOv5s, the pretrained and small version of YOLOv5, which is available for training and use in [43].

In this work, the implementation of YOLO available in [43] was used in conjunction with the free version of the Google Colab platform, which provides a GPU with 15 GB of memory. The training setup was as follows: batch size = 100, epochs = 30, learning rate = 0.01, and size image = 540.

### 4.3. Proposed Architecture

**Figure 5:**
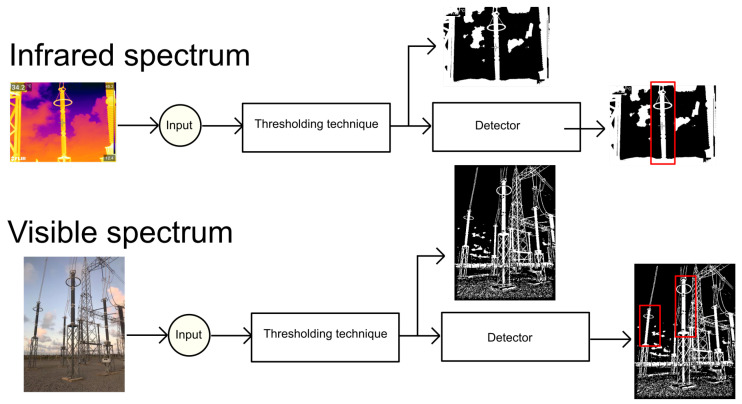
The proposed architecture involves using the same detector for both visible and infrared spectra after the images have been processed by a thresholding technique.

In this work, the detectors are designed to handle both visible and infrared spectra using the architecture shown in Figure 5. First, the image is processed by a thresholding technique, and the detector is then applied. We assume that the range of pixel values of input images is known, so a suitable thresholding technique is used for each kind of image. The thresholding approaches proposed in [44,45] are used for visible and infrared spectra, respectively.

## 5. Database and Preprocessing

This section describes the dataset used to train and validate the VJ and YOLO detectors. Furthermore, the image preprocessing stage is explained.

### 5.1. Synthetic Data

As previously stated, the main investigation in this paper is to evaluate the performance of detectors that were trained with only synthetic positive examples. The reason for that is to investigate the worst-case scenario where no real image of the target objects is available during the training phase.

For this purpose, we used the methodology presented in Section 3 to create synthetic examples of lightning rods and potential transformers. This methodology can be used to render images of electrical equipment with any desired orientation, but only the orientations observed in the GImpSI dataset were explored.

**Figure 6:**
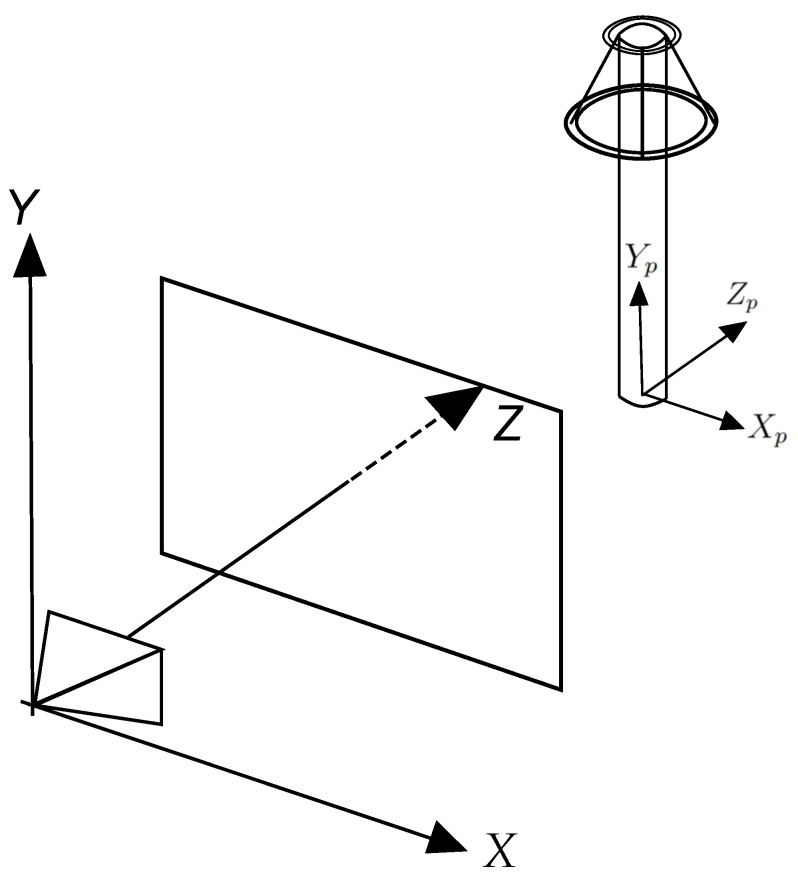
Representation of a electrical equipment and its coordinate system (Xp,Yp,Zp).

Figure 6 illustrates an initial pose (or configuration) of an electrical equipment and its coordinate system ($X_{p},Y_{p},Z_{p}$). Rotations around these three axes are denoted as $\theta_{x}$, $\theta_{y}$, and $\theta_{z}$, respectively. It was empirically noticed that, in the GImpSI dataset, there are only small variations of $\theta_{x}$ and $\theta_{z}$, whereas $\theta_{y}$ was found in a wide distribution of angles. Variations in $\theta_{z}$ were caused by image distortions or camera orientation changes, as well as for $\theta_{x}$ variations, whereas $\theta_{y}$ distribution is chiefly explained by camera rotation around the equipment.

For each equipment, we rendered 1000 synthetic images by randomly sampling the three rotations with the distribution shown in Table 1, where U(a,b) stands for uniform distribution in the range from *a* to *b*, and $N(\mu ,\sigma^{2})$ represents a normal distribution with mean μ and variance $\sigma^{2}$. However, for the VJ detector, the equipment in each rendered image must be clipped and scaled so that its representation becomes a numerical matrix of fixed size. In the case of the lightning rod, the chosen dimensions were arbitrarily set to 95×28, whereas for the potential transformer, they were set to 125×25. This difference in dimensions for the two equipment is due to the difference in their height/width proportions.

**Figure 7:**
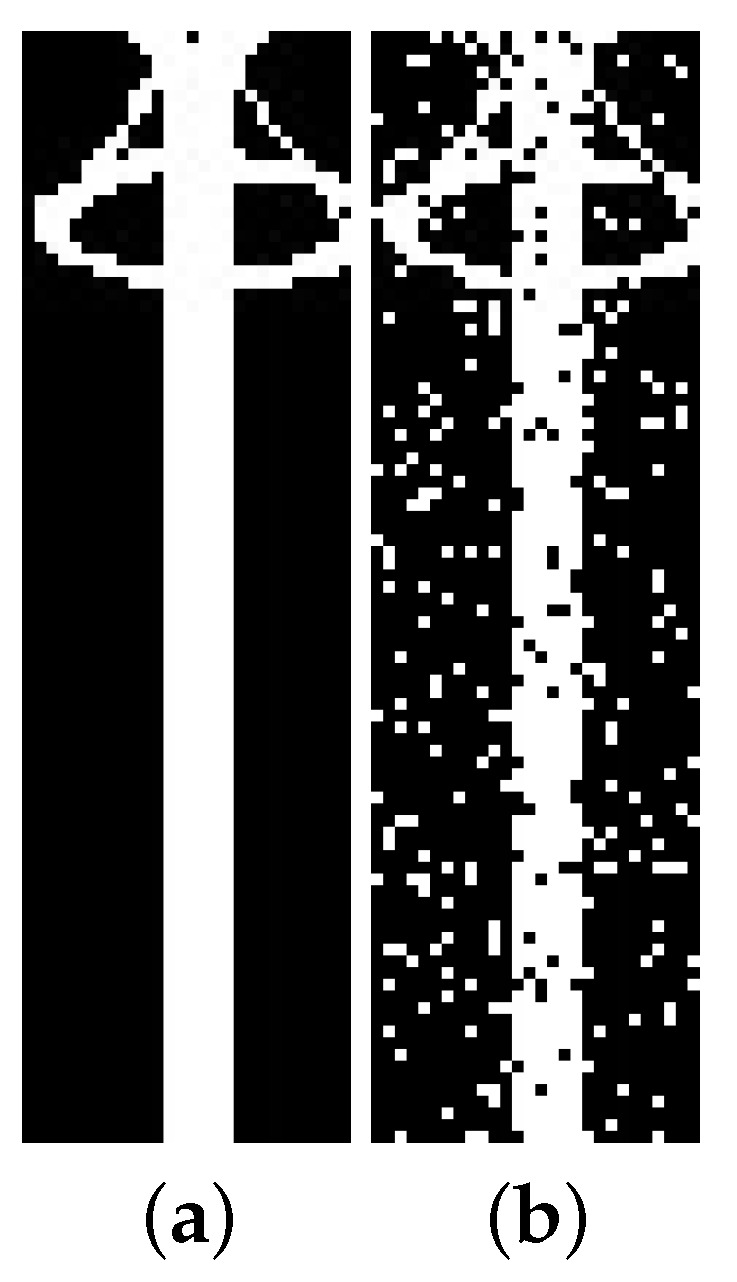
Example of noise insertion, where 10% of the pixels undergo a logical inversion. (**a**) Synthetic lightning rod image before noise inclusion; (**b**) the same image after noise inclusion.

As shown in Figure 4, the background of synthetic images is uniform (i.e., all pixels are black). However, for the detectors to generalize equipment independently of the background, synthetic data should have different backgrounds. Therefore, all 1000 synthetic images of each equipment were duplicated with the addition of noise, as illustrated in Figure 7. The chosen noise insertion methodology was to randomly (and uniformly) select 10% of the pixels and perform a logical inversion on each of them. As a result, two training datasets were created, one for each equipment (target), with 2000 synthetic images per target.

### 5.2. Real Data

**Figure 8:**
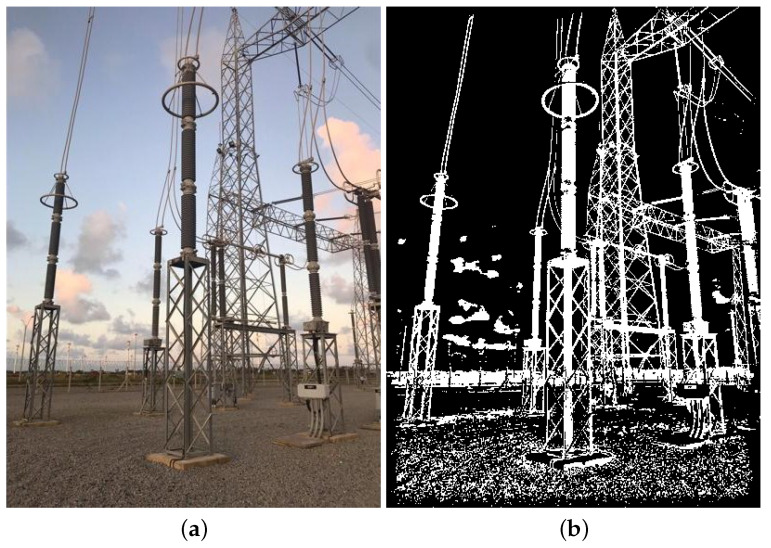
Illustration of the thresholding technique proposed in [44], which is employed in this paper for images in the visible spectrum. (**a**) Color image taken from the GImpSI dataset; (**b**) the same image after its conversion to black and white.

**Figure 9:**
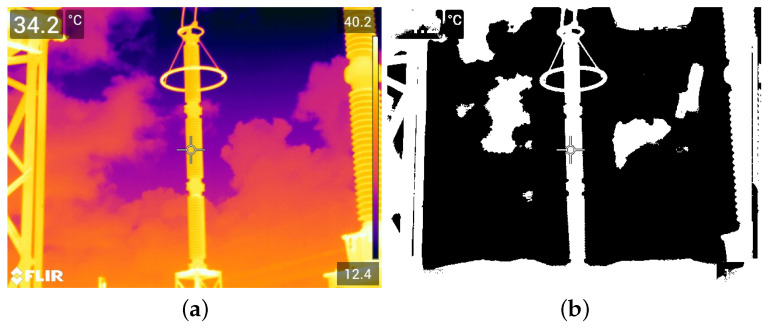
Illustration of the thresholding technique proposed in [45], which is employed in this paper for images in the infrared spectrum. (**a**) Infrared image taken from the GImpSI dataset; (**b**) the same image after conversion to black and white.

The real data used in this paper include images in visible and infrared spectra. Since these are images with pixel attributes represented with several intensity levels (i.e., Red, Green, Blue, Infrared intensities), and because we assume that black-and-white pixels are enough for equipment recognition, all real images are first converted to black and white, or binary images, as the synthetic ones. In that regard, two methods were chosen: the method proposed in [44] for images in the visible spectrum, and another method proposed in [45] for the infrared spectrum. Examples of resulting binary images paired with their corresponding multi-level original versions are shown in Figure 8 and Figure 9, respectively.

**Figure 10:**
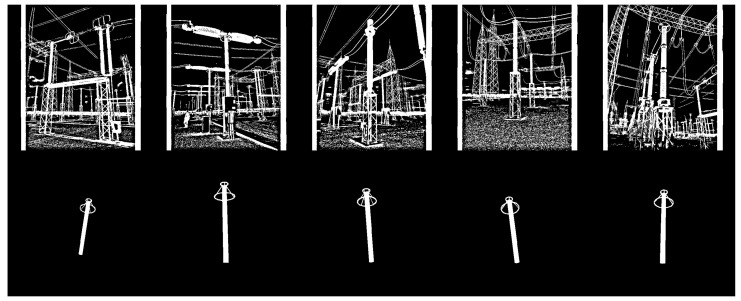
Examples of images from the training dataset for the detectors of lightning rods. The first row consists of non-synthetic negative examples, and the second row consists of synthetic positive examples.

**Figure 11:**
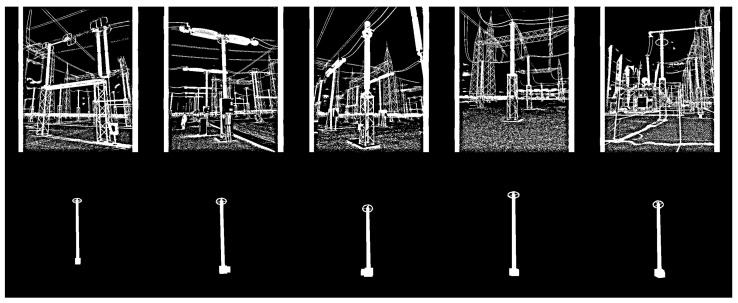
Examples of images from the training dataset for the detectors of potential transformers. The first row consists of non-synthetic negative examples, and the second row consists of synthetic positive examples.

In addition to positive synthesized images of both targets, negative samples were also included in both training datasets. However, unlike the positive instances, negative samples were not synthesized, but taken from the GImpSI dataset, corresponding to images of non-targeted equipment plus noisy backgrounds. Thus, 1000 real color images in the visible spectrum were converted to binary and added to the two training datasets. As a result, two training datasets were created for each equipment, each containing 2000 synthetic examples of the target equipment and 1000 real negative examples. Examples of images from these datasets are shown in Figure 10 and Figure 11 for the lightning rod and potential transformer, respectively.

**Figure 12:**
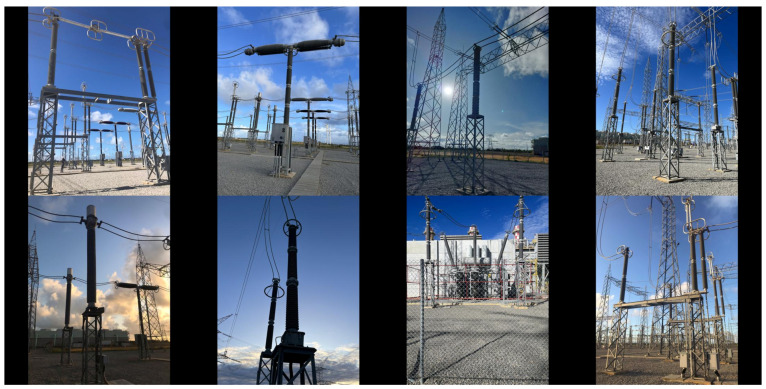
Examples of images in the visible spectrum from the validation datasets $L_{V}$ and $P_{V}$.

**Figure 13:**
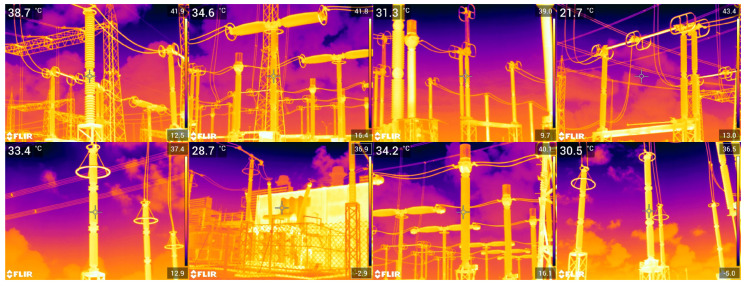
Examples of images in the infrared spectrum from the validation datasets $L_{I}$ and $P_{I}$.

As for validation purpose, four datasets were also built. Specifically, two validation datasets were created for each target equipment: one composed solely of real binary images converted from the visible spectrum, and the other composed of real binary images converted from the infrared spectrum. It is noteworthy that, in many real images, the targeted equipment appears several times. Therefore, $N_{P}$, which stands for the total number of times a target appears in a given set of images, is not a constant proportion of $N_{T}$, the total number of images in that set, as presented in Table 2. Instances of the four validation datasets are shown in Figure 12 (visible) and Figure 13 (infrared).

## 6. Experimental Results

This section presents the performance metrics chosen to evaluate the detectors, the results obtained with the training and validation datasets, and a discussion subsection.

### 6.1. Performance Metrics

Two common performance metrics in detector analysis are recall (*R*), defined in (5), and precision (*P*), which is calculated as in (6):

(5) $$R=\frac{T_{p}}{T_{p}+F_{n}},$$

(6) $$P=\frac{T_{p}}{T_{p}+F_{p}},$$

where $T_{p}$ stands for the number of true positives, or positive examples, that were detected, $F_{n}$ represents the number of positives that were not successfully detected (false negatives), and $F_{p}$ represents the number of incorrectly detected objects (false positives). Recall indicates the percentage of target equipment detected, and precision indicates the number of detected objects that are actual targets.

These two metrics complement each other and depend on the chosen confidence threshold, and for that reason, a third metric, denoted as $F_{1}$, combines *P* and *R* as the harmonic mean of precision and recall, as in (7).

(7) $$F_{1}=2\frac{R\cdot P}{R+P}.$$

The metric $F_{1}$ can be used as a score to identify the best operating point on the precision–recall curve of a detector, considering that precision and recall are equally important. To analyze all operating points, the average precision (AP) can be used, which is defined as the area under the precision–recall curve.

Another important metric is the Intersection over Union (IoU), which compares the two bounding boxes: the ground truth and the prediction made by the detector. IoU is calculated as the ratio between the area of intersection and the area of union of the two bounding boxes. Different thresholds of acceptable IoU yield different values of precision and recall, thereby affecting the values of $F_{1}$ and AP. In this work. IoU ≥ 0.5 is used.

### 6.2. Results with Training Datasets

The performances of the detectors with their training datasets are shown in Table 3. In all cases, the detectors achieved high performance ($F_{1}\geq 0.9$), with YOLO performing better for both targets.

### 6.3. Results with Validation Datasets

**Figure 14:**
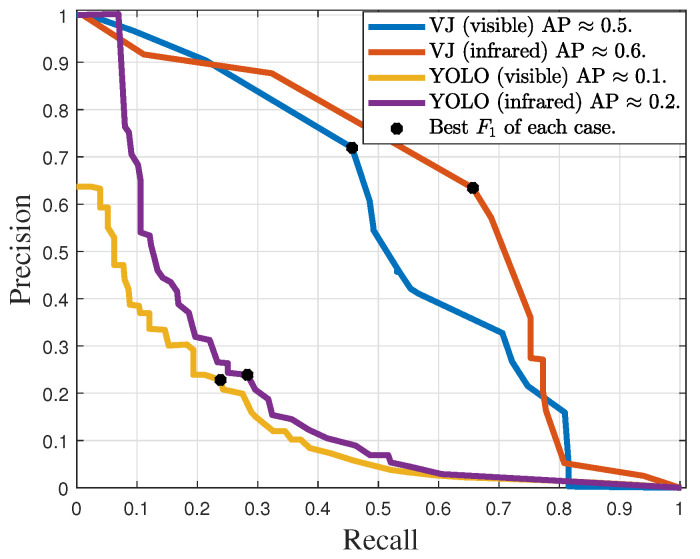
Precision–recall curves of the detectors trained for lightning rod detection with IoU ≥0.5.

The precision–recall curves obtained with VJ and YOLO detectors, trained for lightning rod detection, are shown in Figure 14. YOLO achieved approximate AP values of 0.1 and 0.2, while VJ achieved approximately 0.5 and 0.6. The best operating points of the detectors are summarized in Table 4. The maximum measure $F_{1}$ of the YOLO was smaller than 26%, for both spectra, while the VJ detector obtained 55.8% for images in the visible spectrum, and 60.5% for infrared images.

**Figure 15:**
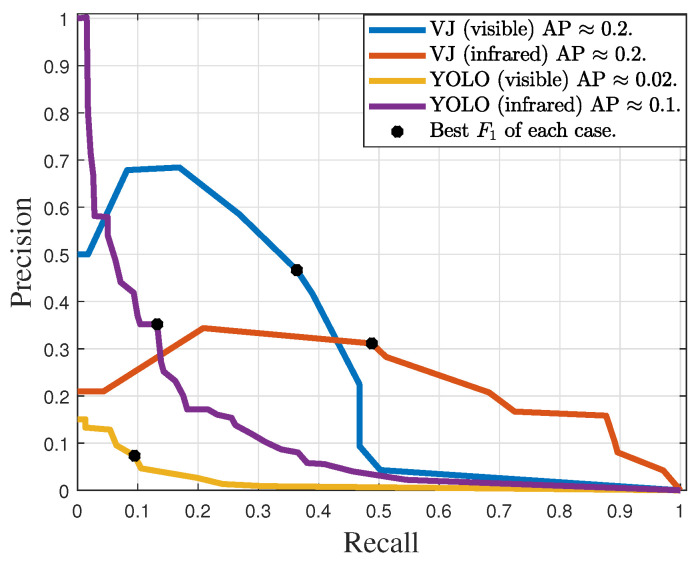
Precision–recall curves of the detectors trained for potential transformer detection with IoU ≥0.5.

Figure 15 shows the precision–recall curves obtained with the VJ and YOLO detectors, trained for potential transformers. YOLO achieved approximate AP values of 0.02 and 0.1, while VJ achieved approximately 0.2 in both cases. The best operating points of the detectors are summarized in Table 4. The maximum measured $F_{1}$ for YOLO was smaller than 20%, while the measured $F_{1}$ values for VJ were 40.9% and 38.0% for images in the visible and infrared spectra, respectively.

### 6.4. Discussion

As shown in Table 4 and Table 5, the $F_{1}$ scores for the VJ detector exceeded twice the highest $F_{1}$ scores achieved by YOLO in all validation datasets. This performance difference between the detectors was also measured in terms of average precision, as shown in Figure 14 and Figure 15. However, as discussed in [46], YOLO typically outperforms the VJ detector. The relatively lower performance exhibited by YOLO may be attributed to the use of only binary images during the training phase, coupled with the fact that all positive examples of the target equipment in the training dataset consisted of synthetic images. Indeed, while the VJ detector is well-fitted to use the restricted geometric information preserved in binary images, the YOLO strongly relies on a retraining step to cope with its huge amount of free parameters to be tuned. Therefore, because this pretraining is done with images where textures and colors are preserved, one may conjecture that YOLO performance is strongly disturbed by the image binarization step. Alas, being a black-box kind of machine learning, the confirmation of this conjecture is not straightforward, and it is beyond the scope of this work.

**Figure 16:**
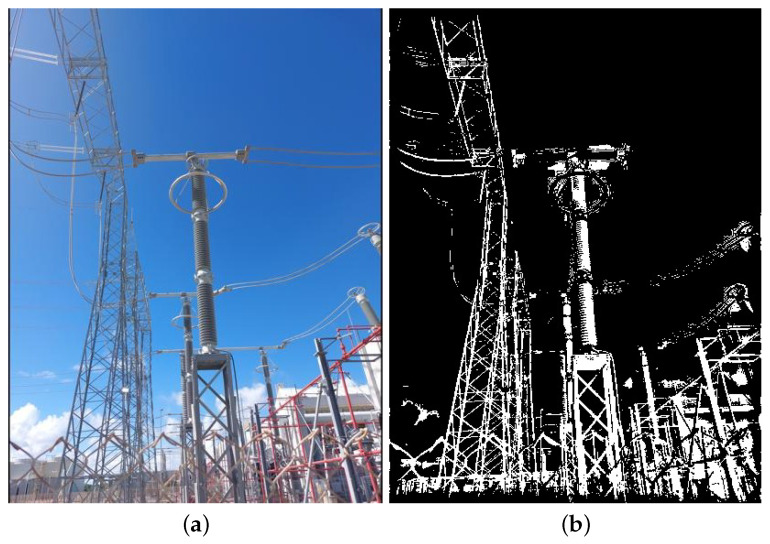
Example of an image in which the lightning rod image was distorted in the thresholding process due to sunlight, and the VJ detector was not able to detect it. (**a**) Color image; (**b**) the same image converted to black and white.

However, regardless of which detector exhibited superior performance concerning the training and validation datasets, the higher $F_{1}$ values experimentally observed seem to corroborate that one may rely solely on synthetic images as positive examples during the training phase for rigid target objects. In this context, the results obtained from the VJ detector suggest that a detector can achieve satisfactory outcomes, even if the $F_{1}$ score for the VJ detector is lower than what is typically achieved when it is trained with color images. Besides the information carried out by the target colors and textures, we also hypothesize that the VJ detector’s performance is somewhat compromised by distortions experienced by electrical equipment images during the thresholding process, particularly distortions induced by sunlight. For instance, in Figure 16, an image is shown where there was a relevant distortion in the lightning rod, to the point that the VJ detector is unable to detect it.

**Figure 17:**
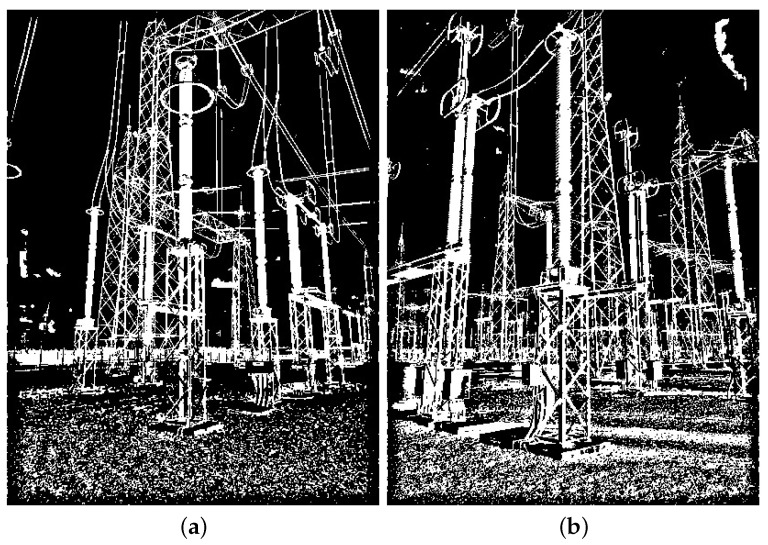
Examples of images in which the VJ detector was not able to detect the targeted equipment, despite the absence of distortion. (**a**) Lighting rod; (**b**) potential transformer.

**Figure 18:**
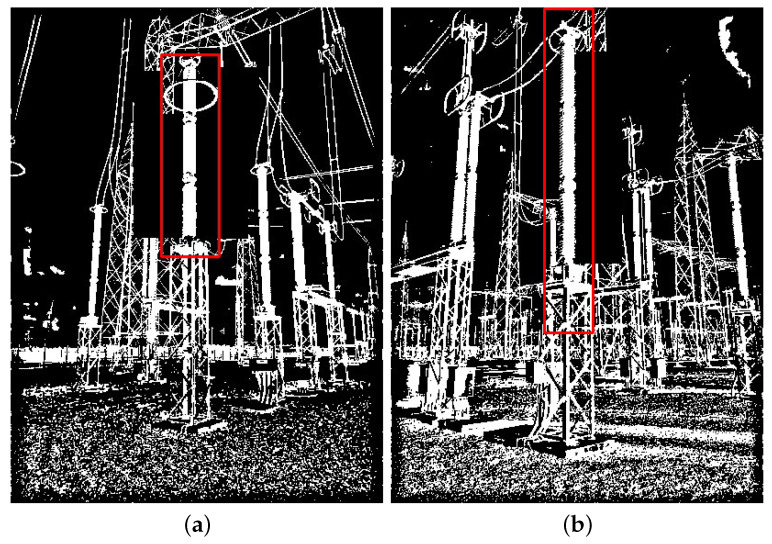
Results of the VJ detector for the same images shown in Figure 17, but with part of the background manually removed. (**a**) Lighting rod; (**b**) potential transformer.

In addition to the distorted images, there were also some images without distortion in which the VJ detector was not able to detect the targeted equipment. In these images, what hindered the detection process were other objects cluttered behind the target. To illustrate this effect, the two images shown in Figure 17 were selected. After the background just around the target was manually cleaned, as in Figure 18, the same VJ detector was able to detect the target.

**Figure 19:**
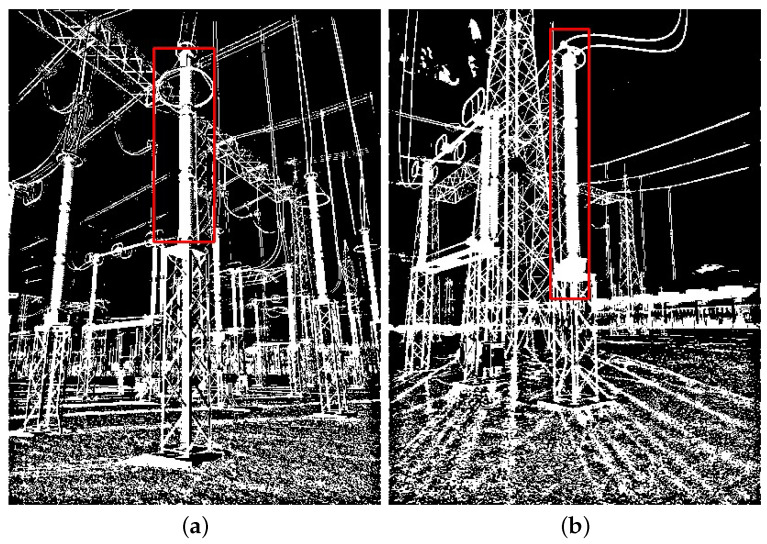
Two examples of images in which the VJ correctly detected targets despite the presence of other objects behind it. (**a**) Lighting rod; (**b**) potential transformer.

It is worth noting that not all objects behind targets hinder detection. In Figure 19, two examples are shown where the detection was successful despite the presence of objects in the target background.

## 7. Conclusions

The main contribution of this work is a case study where two learning machines from different paradigms are assessed when synthetic images are used for training, thus simulating a scenario where an industrial plant is designed but not yet built, and visual targets are rigid bodies whose designs are precisely known. Two classes of power electrical equipment images were arbitrarily chosen for this study. The learning machines selected were the Viola–Jones and the YOLO detectors. The Viola–Jones is a relevant instance of explainable and efficiency-tuned methods, whereas the YOLO is a popular machine learning approach in the context of the so-called deep-learning paradigm. Both machine learning models were trained using synthetic images of the mentioned two types of equipment as targets for detection. Additionally, non-synthetic instances of images not related to the targets were included in the training dataset, all of which had previously undergone binarization, meaning that they were converted into black and white. For each of the targeted equipment types, namely lightning rods and potential transformers, both detectors underwent tests using binarized versions of non-synthetic images obtained from the GImpSI database, encompassing images from both the visible and infrared spectra. It is worth highlighting that the binarization process facilitates the use of the same detector for both spectra and also simplifies the rendering process, as only the geometrical descriptions of the devices were required.

For all the spectra and devices tested, the $F_{1}$ measure for YOLO was smaller than 26%, while the $F_{1}$ measure for the Viola–Jones detector ranged approximately from 38% to 61%. YOLO may have performed poorly compared to the VJ detector because it was pre-trained on color images and may not have been able to fully learn to generalize detection in black-and-white images, even after being retrained on a dataset of black-and-white images.

By contrast, the performance of the VJ detector indicates that a detector trained with synthetic images of rigid equipment can achieve useful results. Furthermore, it was observed that some images not detected by the VJ detector presented strong distortion caused by sunlight. Thus, the performance of the detector can be improved if a better binarization method is used, in terms of robustness to sunlight/shadow effects.

In addition to the distortion caused by sunlight, complex and cluttered backgrounds also affected the performance of the VJ detector. This was illustrated through images where the VJ detector only succeeded after a portion of the background was manually removed. The difficulty of finding this type of equipment was already expected because, during the synthesis stage, objects were free from structured background noise. Indeed, the only noise simulated during training was a logical inversion in 10% of the pixels of half of the synthetic images used in the training.

Thus, one should expect further improvement in equipment detection through the synthesis of images with more representative background noise, including non-targeted types of equipment and/or objects and textures typically found around that kind of industrial environment. However, even results obtained so far seem to corroborate the belief that rigid bodies, especially those whose precise descriptions are easily available (such as industrially manufactured equipment), are indeed strong candidates for this kind of approach, where machine learning methods can be adjusted even without any real images of the equipment.

Although this paper does not focus on enhancing detector performance in a general sense, it does provide valuable insights into the utilization of prior knowledge concerning rigid bodies for fine-tuning operational detectors in the absence of genuine target images.

In terms of future research, our objectives include experimenting with the synthesis of equipment images that incorporate additional objects in the background and implementing thresholding techniques that demonstrate increased resilience to issues related to sunlight and shadow effects.

## Figures and Tables

**Table 1:** Random distribution of rotation angles used to render equipment images. Angle values are given in degrees.

Variable	$\theta_{x}$	$\theta_{y}$	$\theta_{z}$
Distribution	U(−45,−10)	U(−180,180)	N(0,25)

**Table 2:** Composition of the four validation datasets. $N_{T}$ represents the total number of images, and $N_{P}$ represents the number of times that the target equipment appears in these images.

Validation Dataset	Target	Spectrum	$N_{T}$	$N_{P}$
$L_{V}$	Lighting rod	Visible	845	309
$L_{I}$	Lighting rod	Infrared	318	159
$P_{V}$	Potential Transformer	Visible	845	231
$P_{I}$	Potential Transformer	Infrared	396	196

**Table 3:** Performances of the VJ and YOLO detectors in the training datasets with IoU ≥0.5.

Detector	Target	*R*	*P*	$F_{1}$
VJ	Lighting rod	0.841	0.976	0.903
YOLO	Lighting rod	1.0	0.993	0.996
VJ	Potential Transformer	0.909	0.974	0.940
YOLO	Potential Transformer	1.0	1.0	1.0

**Table 4:** Performances of the VJ and YOLO detectors trained for lightning rod detection with IoU ≥0.5. Dataset $L_{V}$ is composed of images in the visible spectrum, and $L_{I}$ is composed of infrared images.

Detector	Validation Dataset	*R*	*P*	$F_{1}$
VJ	$L_{V}$	0.456	0.719	0.558
YOLO	$L_{V}$	0.238	0.228	0.233
VJ	$L_{I}$	0.657	0.634	0.605
YOLO	$L_{I}$	0.283	0.239	0.259

**Table 5:** Performances of the VJ and YOLO detectors trained to detect potential transformers with IoU ≥0.5. Dataset $P_{V}$ is composed of images in the visible spectrum, whereas $P_{I}$ is composed of infrared images.

Detector	Validation Dataset	*R*	*P*	$F_{1}$
VJ	$P_{V}$	0.364	0.467	0.409
YOLO	$P_{V}$	0.095	0.073	0.082
VJ	$P_{I}$	0.488	0.311	0.380
YOLO	$P_{I}$	0.132	0.352	0.192

## Data Availability

The data that support the findings of this study are available from the corresponding author, upon reasonable request.

## Abbreviations

The following abbreviations are used in this manuscript:

CNN	Convolutional Neural Network
VJ	Viola–Jones
YOLO	You Only Look Once
