# Correction: Santos et al. Performance Assessment of Object Detection Models Trained with Synthetic Data: A Case Study on Electrical Equipment Detection. *Sensors* 2024, *24*, 4219

**DOI:** 10.3390/s25154801

**Published:** 2025-08-04

**Authors:** David O. Santos, Jugurta Montalvão, Charles A. C. Araujo, Ulisses D. E. S. Lebre, Tarso V. Ferreira, Eduardo O. Freire

**Affiliations:** 1 Department of Electrical Engineering, Federal University of Campina Grande, Campina Grande 58401-490, Brazil; david.oliveira.santos@ee.ufcg.edu.br; 2 INESC P&D Brasil, Santos 11055-300, Brazil; jmontalvao@academico.ufs.br (J.M.); efreire@conicet.gov.ar (E.O.F.); 3 Department of Electrical Engineering, Federal University of Sergipe, São Cristóvão 49100-000, Brazil; 4 Electrical Operation, Eneva S.A., Barra dos Coqueiros 49140-000, Brazil; charles.cordeiro@eneva.com.br (C.A.C.A.); ulisses.lebre@eneva.com.br (U.D.E.S.L.); 5 National Council of Scientific and Technical Research—CONICET, Godoy Cruz, Buenos Aires 2290, Argentina

The changes are described below:

1. INESC P&D Brasil was included as an entity to which several authors are affiliated;
2. David O. Santos, Jugurta Montalvão, Tarso V. Ferreira, and Eduardo O. Freire were linked to INESC P&D Brasil;
3. The author to whom correspondence should be sent was changed to Tarso V. Ferreira <tarso.vilela@inescbrasil.org.br>.

Despite these difficulties, there have already been remarkable achievements in object detection [1].

The authors state that the scientific conclusions are unaffected. This correction was approved by the Academic Editor. The original publication has also been updated.

## References

[1] Santos D.O., Montalvão J., Araujo C.A.C., Lebre U.D.E.S., Ferreira T.V., Freire E.O. (2024). Performance Assessment of Object Detection Models Trained with Synthetic Data: A Case Study on Electrical Equipment Detection. Sensors.

